# Supplementary material for: Explaining the flaws in human random generation as local sampling with momentum
Source: PLoS Comput Biol. 2024 Jan 5;20(1):e1011739. doi: 10.1371/journal.pcbi.1011739 (PMC10796055; doi:10.1371/journal.pcbi.1011739)
Supplement: S1 Text — (PDF) [file pcbi.1011739.s001.pdf]

S1 Text Additional Exploratory Plots

S1.1 First Half-Second Half QQ-Plots

Because in Experiment 1 participants sampled from an unknown distribution, we evaluated whether the empirical distribution they produced changed through time. We divided participants' sequences into two halves, and plotted empirical QQ plots to examine whether their distributions were the same in both halves (Fig A). We also computed two-sample Kolmogorov-Smirnov tests comparing the two subsequences. We found a significant difference in ten (25%) sequences. However, we found that it was equally likely for these shifts to occur in both conditions (with five significant results in each condition). We also found that in 5 sequences the empirical distribution function of the first half exceeded that of the second, while in the other five it was the the empirical distribution function of the second that exceeded that of the first.

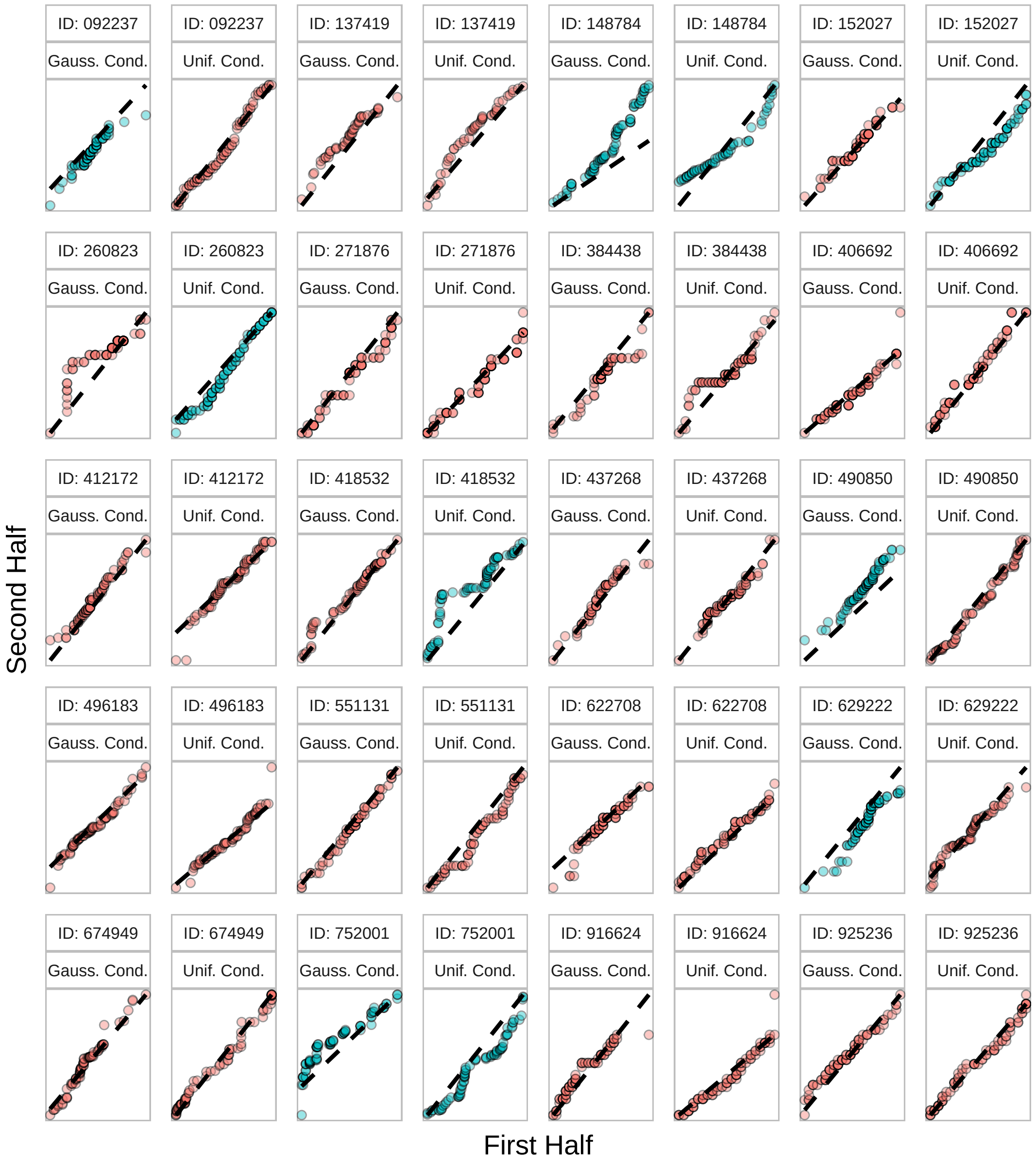

Fig A: Empirical QQ-Plots between a participant's first half and second half of the uttered sequence. Blue plots represent sequences with a significant Kolmogorov-Smirnov result comparing the two halves.

S1.2 Autocorrelation plots

Another useful exploratory plot are the autocorrelations at different lags of participants' sequences and the different models (Fig B), where we see that participants' autocorrelations are large when the lag is low, but decrease more steeply than all the proposed models.

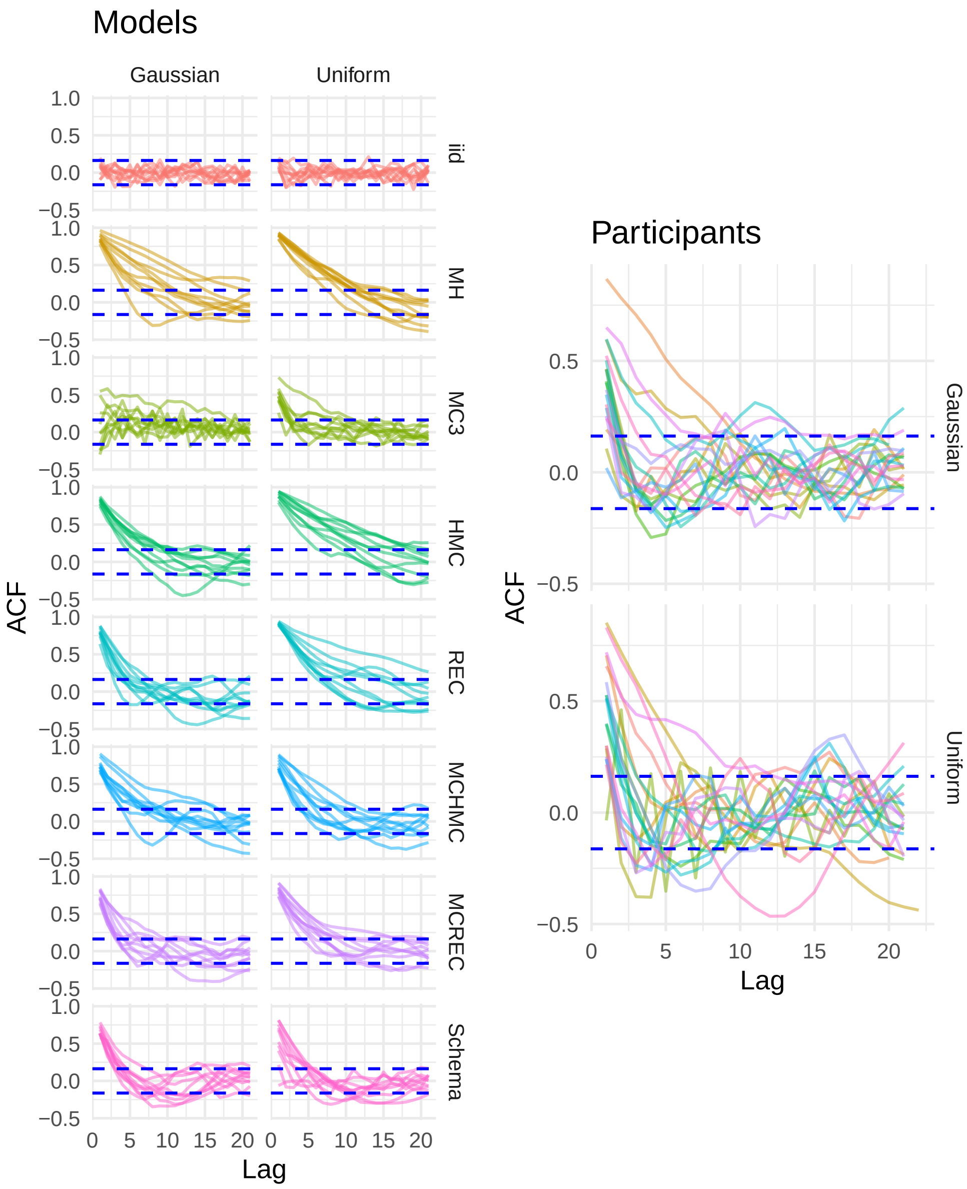

Fig B: Autocorrelation plots for sequences in the Gaussian and Uniform conditions of Experiment 1, for (A) the ten best fitting sequences of every model and (B) participants (each participant one color). Dashed blue lines represent 95% confidence intervals.

S1.3 Fitted-Empirical QQ-Plots

We also evaluated the fit of the distribution to participants' sequences by plotting a QQ Plot between the empirical quantiles, and the theoretical quantiles defined by the best fitting distribution (Beta in the uniform condition, Normal in the gaussian condition; Fig C). This reveals that fit was generally good, but could have been better for some participants. To ensure that modeling results were not caused by poor goodness of fit (which influences the local samplers and *iid* model), we carried out a Kolmogorov-Smirnov test to evaluate the fit of the sequences (we highlight significant results in the figure), and re-analyzed the results after excluding participants with a significant result. This exclusion does not alter our results, with this subset of the data showing strong support for local sampling models over the next best model ($BF_{10} = 1.3 \times 10^{11}$), and with very strong evidence in favor of the features we proposed: multiple chains ($BF_{10} = 3.0 \times 10^{13}$), gradient-based proposals ($BF_{10} = 4.5 \times 10^{12}$) and momentum ($BF_{10} = 4.4 \times 10^{6}$).

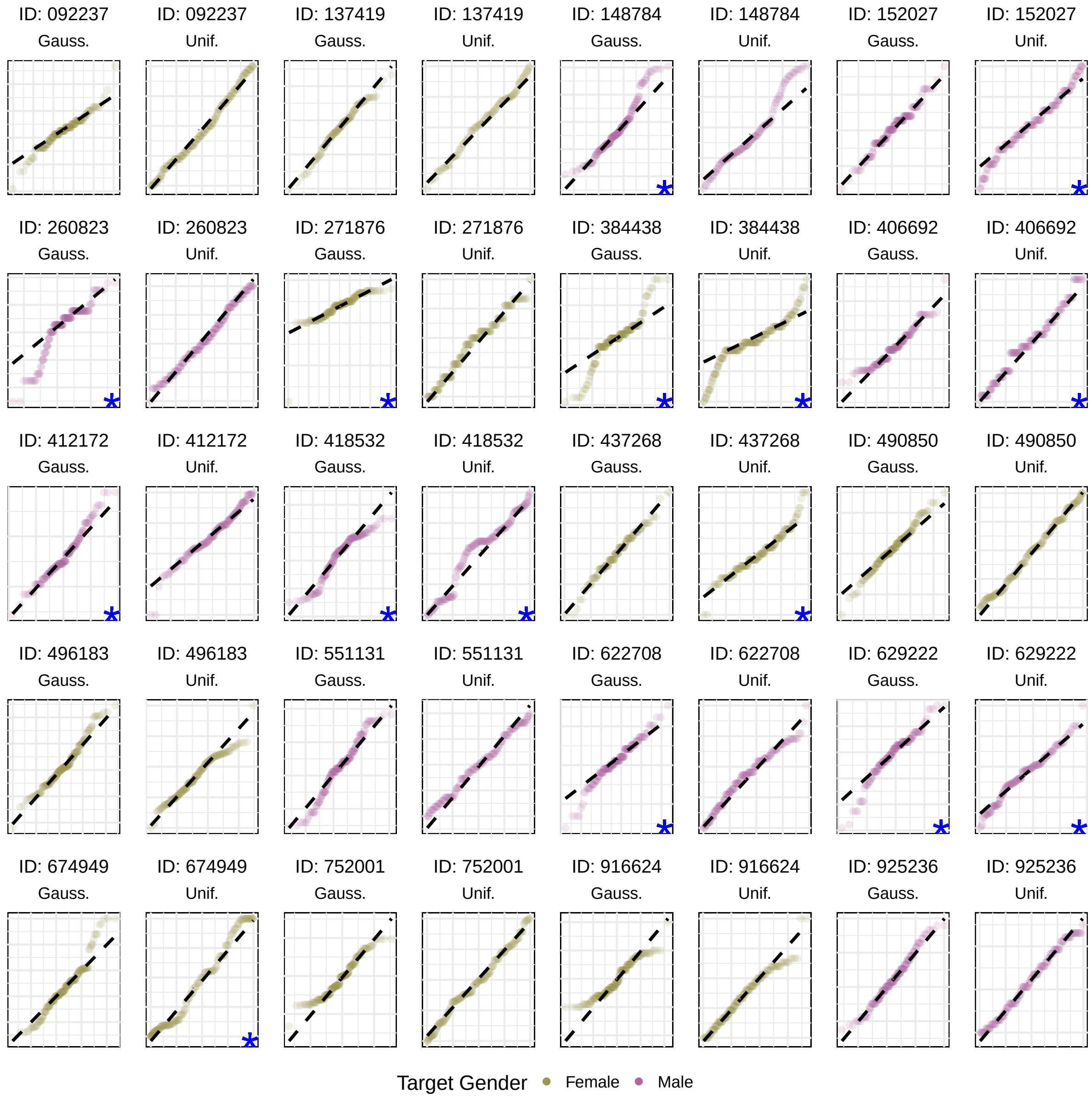

Fig C: Theoretical-Empirical QQ Plots between the canonical distribution fit to each participant's sequence and the empirical sequence, and evaluated at each sample. Panels marked with an asterisk are those where a Kolmogorov-Smirnov test was significant (i.e. the null hypothesis that the empirical sequence and the theoretical fit are the same distribution was rejected). Color represents whether the participant sampled male or female heights.
