# Supplementary material for: Explaining the flaws in human random generation as local sampling with momentum
Source: PLoS Comput Biol. 2024 Jan 5;20(1):e1011739. doi: 10.1371/journal.pcbi.1011739 (PMC10796055; doi:10.1371/journal.pcbi.1011739)
Supplement: S2 Text — (PDF) [file pcbi.1011739.s002.pdf]

S2 Text Condition effects

Experiment 1

The differences between participants' measures and the expectation from reshuffled sequences differed due to Condition for *Distances* ($t(5471.89) = 7.93$, $p < .001$, $d = 0.18$, $BF_{10} = 6.8 \times 10^4$), *Repetitions* ($Z = 4.63$, $p < .001$, $d = 0.45$, $BF_{10} = 473$) and *Adjacencies* ($Z = -6.94$, $p < .001$, $d = -0.39$, $BF_{10} = 4.1 \times 10^4$), with ambiguous evidence for *Turning Points* ($Z = -2.44$, $p = .01$, $d = -0.11$, $BF_{10} = 1/4$).

These, however, were only differences in the degree of deviation and not the direction or significance of effects, as revealed by posthoc tests: in both conditions, participants had higher *Adjacencies* (Uniform: Obs. = .21, Exp. = .06, Z = 6.23, $p < .001$, $d = 1.09$, $BF_{10} = 933$; Gaussian: Obs. = .20, Exp. = .09, $Z = 5.02$, $p < .001$, $d = 0.79$, $BF_{10} = 206$), lower *Repetitions* (Uniform: Obs. = .008, Exp. = .034, $Z = -3.24$, $p = .001$, $d = -1.22$, $BF_{10} = 69$; Gaussian: Obs. = .02, Exp. = .05, $Z = -2.30$, $p = .02$, $d = -0.63$, $BF_{10} = 3$), lower *Turning Points* (Uniform: Obs. = .49, Exp. = .66, $Z = -11.11$, $p < .001$, $d = -0.59$, $BF_{10} = 5.2 \times 10^7$; Gaussian: Obs. = .43, Exp. = .65, $Z = -6.3$, $p < .001$, $d = -0.69$, $BF_{10} = 2.8 \times 10^3$) and smaller *Distances* (Uniform: Obs. = 11.90, Exp. = 22.08, $t(18.93) = -3.71$, $p = .001$, $d = -0.45$, $BF_{10} = 14$; Gaussian: Obs. = 8.68, Exp. = 14.76, $t(19.02) = -2.56$, $p = .019$, $d = -0.36$, $BF_{10} = 1$).

Experiment 2

No difference between conditions was found for either *Repetitions* ($Z = 0.95$, $p = .34$, $d = 0.26$, $BF_{10} = 1/11$) nor *Turning Points* ($Z = -1.78$, $p = .08$, $d = -0.16$, $BF_{10} = 1/9$). Participants in the one-dimensional condition had higher *Adjacencies* and lower *Distances* than *iid*, but participants in the two-dimensional condition did not: Evidence for a difference between conditions was decisive for both *Adjacencies* ($Z = -4.93$, $p < .001$, $d = -0.55$, $BF_{10} = 274$) and *Distances* ($t(38.00) = 5.94$, $p < .001$, $d = 0.48$, $BF_{10} = 1.1 \times 10^3$).
