# Supplementary material for: Explaining the flaws in human random generation as local sampling with momentum
Source: PLoS Comput Biol. 2024 Jan 5;20(1):e1011739. doi: 10.1371/journal.pcbi.1011739 (PMC10796055; doi:10.1371/journal.pcbi.1011739)
Supplement: S3 Text — (PDF) [file pcbi.1011739.s003.pdf]

S3 Text Order Effects

Experiment 1

First, we analyzed possible effects of *Order*. We found a credible main effect of *Order* for *Repetitions* ($Z = 3.84$, $p < .001$, $d = 0.44$, $BF_{10} = 31$), with participants having higher values in the second sequence, but no credible *Order* × *Condition* interaction ($Z = -0.31$, $p = .76$, $d = -0.16$, $BF_{10} = 1/6$). The same was true for *Distances*, with participants having higher values in their first sequence: the main effect was credible ($t(5470.94) = 4.61$, $p < .001$, $d = 0.11$, $BF_{10} = 18$) but the interaction was not ($t(17.98) = -1.64$, $p = .12$, $d = -0.40$, $BF_{10} = 1/5$). We found evidence against the main effect and against the interaction for *Adjacencies* (Main Effect: $Z = -0.87$, $p = .39$, $d = -0.05$, $BF_{10} = 1/47$; Interaction: $Z = -0.46$, $p = .65$, $d = -0.14$, $BF_{10} = 1/11$) and *Turning Points* (Main Effect: $Z = -2.30$, $p = .02$, $d = -0.11$, $BF_{10} = 1/6$; Interaction: $Z = 0.03$, $p = .98$, $d < 0.01$, $BF_{10} = 1/33$).

When separating the data into the two values of *Order*, we found that these differences were in degree of deviation only, but that in both orders participants followed the same deviations from *iid* as reported in the main text (Table A).

Measure	Order	Obs.	Exp.	Statistic	p	d	BF_{10}
Repetitions	1st	.013	.04	$Z = -3.23$	.001	−1.14	51
Repetitions	2nd	.012	.04	$Z = -2.41$	.02	−0.73	4
Distances	1st	11.44	20.77	$t(17.97) = -3.72$	.002	−0.42	8
Distances	2nd	9.15	16.06	$t(17.99) = -2.85$	.01	−0.42	2

Table A: Results of four models comparing participant's *Repetitions* and *Distances* to the expectation derived from reshuffled sequences. People's values are lower than the expectation despite the order effects.

Experiment 2

We found a significant but not credible main effect of *Order* for *Repetitions* ($Z = -2.79$, $p < .01$, $d = -0.14$, $BF_{10} = 1/2$), while evidence for a main effect of *Order* was not significant for *Adjacencies* ($Z = 0.06$, $p = .96$, $d = 0.001$, $BF_{10} = 1/204$), *Turning Points* ($Z = -1.06$, $p = .29$, $d = -0.02$, $BF_{10} = 1/106$) or *Distances* ($t(2.6 \times 10^4) = -1.75$, $p = .08$, $d = -0.02$, $BF_{10} = 1/90$). We found a credible *Order* × *Condition* interaction in some measures: this effect was credible for *Repetitions* ($Z = -4.59$, $p < .001$, $d = -0.23$, $BF_{10} = 49$) and *Distances* ($t(2.6 \times 10^4) = 4.20$, $p < .001$, $d = 0.05$, $BF_{10} = 14$), and constituted anecdotal evidence for *Adjacencies* ($Z = -3.37$, $p < .001$, $d = -0.07$, $BF_{10} = 2$). We found evidence against an interaction for *Turning Points* ($Z = -0.34$, $p = .73$, $d = -0.008$, $BF_{10} = 1/168$). Posthoc analyses suggest that these moderating effects were small in size, and do not change the general conclusions of whether people deviated from *iid* sampling or whether they did so differently in each dimension, in any of the three measures where these effects are significant, as shown in Table B.

Measure	Condition	Order	Obs.	Exp.	Statistic	p	d	BF_{10}
Repetitions	1D	1st	.035	.150	$Z = -7.62$	< .001	−1.59	8.1×10^4
Repetitions	1D	2nd	.037	.152	$Z = -7.01$	< .001	−1.46	2.6×10^4
Repetitions	2D	1st	.042	.146	$Z = -4.72$	< .001	−1.01	1.0×10^3
Repetitions	2D	2nd	.035	.148	$Z = -5.77$	< .001	−1.26	3.8×10^3
Adjacencies	1D	1st	.46	.28	$Z = 5.18$	< .001	0.58	441.21
Adjacencies	1D	2nd	.48	.29	$Z = 5.21$	< .001	0.62	634
Adjacencies	2D	1st	.59	.55	$Z = 1.58$	.12	0.11	1/9
Adjacencies	2D	2nd	.57	.56	$Z = 0.77$	.44	0.05	1/23
Distances	1D	1st	2.26	2.71	$t(19.05) = -4.76$	< .001	−0.30	19
Distances	1D	2nd	2.18	2.70	$t(19.06) = -5.19$	< .001	−0.35	44
Distances	2D	1st	1.46	1.37	$t(19.01) = 0.90$	.38	0.11	1/13
Distances	2D	2nd	1.48	1.36	$t(19.01) = 1.52$	.15	0.15	1/9

Table B: Results of models in each possible *Order* × *Dimension* subset of the data in Experiment 2. As stated in the main text, *Repetitions* were lower than *iid* sampling, whereas *Adjacencies* and *Distances* were higher and lower respectively in the one-dimensional condition, but not the two-dimensional condition. These conclusions were not affected by whether the sequence was the 1st or 2nd sequence participants uttered.
