# Supplementary material for: Explaining the flaws in human random generation as local sampling with momentum
Source: PLoS Comput Biol. 2024 Jan 5;20(1):e1011739. doi: 10.1371/journal.pcbi.1011739 (PMC10796055; doi:10.1371/journal.pcbi.1011739)
Supplement: S4 Text — (PDF) [file pcbi.1011739.s004.pdf]

S4 Text Other pre-registered analyses

In Experiment 1, we pre-registered additional analyses that we do not report in the main text.

Randomness Beliefs

After they generated both sequences, we provided participants with a questionnaire aimed to test their beliefs on randomness. We collected the Gambling Fallacies Measure [1, GFM], and also asked participants which of two sequences of coin tosses was more likely to come from an unbiased coin, following Lopes and Oden [2, the biased coins having too many repetitions in some questions and too many alternations in others]. We evaluated whether the deviations from *iid* reported in the main text depended on randomness beliefs by running models

$$ObservedValue = \beta_0 + \beta_1 \times RandomnessBeliefMeasure + 1 \times iidValue + u_i$$

for both the GFM and the Coin Bias measures, and evaluating whether β_1 differed from 0. Randomness beliefs did not modify the obtained measures in any case (see Table A).

Measure	Randomness Beliefs Measure	Statistic	p	BF_{10}
Repetitions	GFM	$Z = -2.31$	.02	1
Adjacencies	GFM	$Z = -1.40$	.16	1/5
Turning Points	GFM	$Z = 0.52$	.60	1/30
Distances	GFM	$t(18.00) = 0.55$	.59	1/16
Repetitions	Coin Bias	$Z = 0.01$	.99	1/7
Adjacencies	Coin Bias	$Z = 0.29$	.77	1/11
Turning Points	Coin Bias	$Z = -1.56$	.12	1/15
Distances	Coin Bias	$t(17.99) = 0.91$	.37	1/12

Table A: Results of eight models evaluating whether participants' deviations from *iid* sampling depended on two measures of beliefs on randomness. We found evidence against the fact that beliefs on randomness influenced the deviations from randomness found

Target Gender

Because we allocated participants to sample heights from either men on women, we also inspected whether target gender influenced the observed patterns:

$$ObservedValue = \beta_0 + \beta_1 \times TargetGender + 1 \times iidValue + u_i$$

We found no differences due to gender (see Table B).

Measure	Statistic	p	d	BF_{10}
Repetitions	$Z = 0.395$	.69	0.20	1/6
Adjacencies	$Z = -0.58$	.56	-0.18	1/10
Turning Points	$Z = -0.13$	.89	-0.02	1/34
Distances	$t(17.98) = -0.21$	.84	-0.05	1/18

Table B: Results of four models evaluating whether participants' deviations from *iid* sampling depended on the gender they had been allocated to

Alternative Widths for Turns at the Center

We analyzed whether people made fewer turns at the center for a wider range of center widths: while in the main text we report a central region corresponding to 25% of the mass for each target distribution of Experiment 1, here we show that the same qualitative results appear if focusing on the central 20% or 30% of the distribution. Irrespective of the center definition, participants made fewer turns at the center of the distribution than reshuffled sequences (For a narrower center: Obs. = .51, Exp. = .40 , $Z = -3.64$, $p < .001$, $d = -0.36$, $BF_{10} = 7$; for a wider center: Obs. = .52, Exp. = .41, $Z = -3.95$, $p < .001$, $d = -0.36$, $BF_{10} = 10$).

References

1. Williams R. Reliability and Validity of Four Scales to Assess Gambling Attitudes, Gambling Knowledge, Gambling Fallacies and Ability to Calculate Gambling Odds. Unpublished technical report. 2003

2. Lopes LL and Oden GC. Distinguishing between Random and Nonrandom Events. Journal of Experimental Psychology: Learning, Memory, and Cognition. 1987; 13:392
