# Supplementary material for: Explaining the flaws in human random generation as local sampling with momentum
Source: PLoS Comput Biol. 2024 Jan 5;20(1):e1011739. doi: 10.1371/journal.pcbi.1011739 (PMC10796055; doi:10.1371/journal.pcbi.1011739)
Supplement: S5 Text — (PDF) [file pcbi.1011739.s005.pdf]

# S5 Text   Model Details

## Schema model

As stated in the main text, we used the schema model found in Cooper [1] as a stand-in for schema accounts more generally, as it is the only computational implementation of a schema account. The model is available at http://www.ccnl.bbk.ac.uk/models. We implement their code exactly, with the only modification being that we modify the response set size to match the size of the space in our tasks, as described in the main text (This variable is called *RESPONSE_SET_SIZE* in the original author's code).

The model consists of several functional components, divided into buffers that store information and processes that manipulate information and read and write it into those buffers (see Fig A). The *Schema Network* buffer contains the set of possible schema $(-5, \ldots, +5)$, one of which is active (except at the start of the task). *Apply Set* reads the *Schema Network* for the active schema (e.g., +3) as well as *Working Memory*, where the last response is stored (e.g., 5), and uses these to produce a new item (e.g., 8) and writes it in the *Response Buffer* (if the response is higher than the response range, it will apply the modulo operation; e.g., 8 would become 1 in Experiment 1). *Generate Response* consults the *Response Buffer* and produces the response, and updates the contents of both *Working Memory* and *Response Buffer*. This procedure is regulated by two supervisory processes (shaded in Fig A). If there is ever no active schema, *Task Control* chooses a schema at random and records it on the *Schema Network*, considering previously used schema (in *Working Memory*), and with each schema having different associated probabilities. *Monitoring* consults the *Response Buffer* where potential items are stored and vets if it is sufficiently random by comparing it to previous responses in *Working Memory*. If not, it deletes the response from the buffer and deselects the active schema.

Besides the eleven fixed weights that each schema has associated, the model's performance is governed by five free parameters: *Memory Decay Time*, which determines the maximum number of iterations a previously-used schema can remain in memory (at which point it becomes unselected without the influence of *Monitoring*); *Memory Update Efficiency*, which determines the probability that the updating the contents of working memory by *Generate Response* will be successful; *Monitoring Efficiency*, which determines the probability that the randomness of the uttered item will be checked by the *Monitoring* process; *Switch Rate*, which determines the probability of switching schemas after each response; and *Temperature*, which softens the distribution of probabilities given to schemas via the softmax function (i.e. the probability of selecting a schema $S_i$ with weight $w_i$ is $\frac{exp(w_i/\tau)}{\sum_j exp(w_j/\tau)}$, where $\tau$ is the temperature parameter).

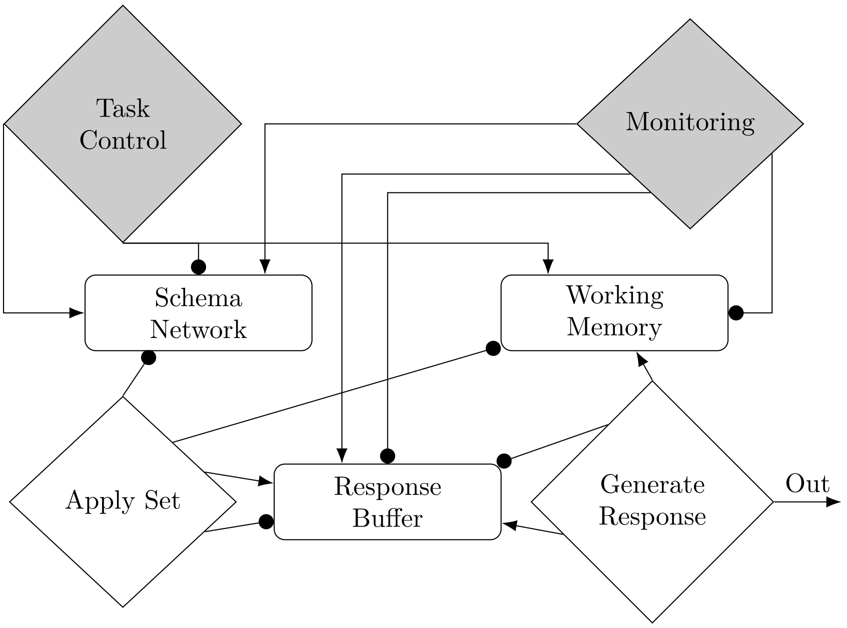


Fig A: Cooper's [1] schema model. Diamond shapes represent processes that manipulate information, while rounded rectangles represent buffers. Standard arrowheads represent write operations, while circle arrowheads represent read operations. The two supervisory processes are shaded. Figure adapted from Cooper [1].

# Local Sampling Models

In the main text, we briefly described the six local sampling algorithms used in the model comparison; we expand on these here. As stated there, these algorithms work by initializing a Markov chain at a random point, then having the chain transition between points in a way that guarantees that, after sufficient iterations, the distribution of registered states in the chain will approximate the true posterior distribution. In our simulations, samplers' starting points were determined by drawing a random sample from the target distribution.

## Metropolis Hastings

After the Markov chain, $\theta$, is initialized at a random point $\theta^1$, the Metropolis-Hastings algorithm proceeds iteratively as follows: in each iteration, it first proposes a random new location $\theta'$ on each iteration by updating the current state with the addition of random normal noise: $\theta' \leftarrow \theta^{n-1} + N(0, \sigma)$, where $n$ is the current iteration and $\sigma$ is a free parameter. Then, it chooses whether to transition to $\theta'$ or not stochastically, with probability $\min\{1, exp\{\mathcal{L}(\theta') - \mathcal{L}(\theta^{n-1})\}\}$, where $\mathcal{L}(\cdot)$ is the log probability density function of the target distribution (see Algorithm 1). We assume a uniform prior on $\sigma$, $\sigma \sim U[0, 50]$.

**Algorithm 1** Metropolis Hastings

**procedure** METROPOLIS HASTINGS($\theta^1$, $\mathcal{L}$, $N$, $\sigma$)
    **for** n=2 to N **do**
        Set $\theta' \leftarrow \theta^{n-1} + N(0, \sigma)$ ▷ Set Proposal
        Set $\alpha \leftarrow exp\{\mathcal{L}(\theta') - \mathcal{L}(\theta^{n-1})\}$
        Sample $u \sim U[0, 1]$
        **if** $u \leq \min\{1, \alpha\}$ **then** ▷ Stochastic transition
            Set $\theta^n \leftarrow \theta'$
        **else**
            Set $\theta^n \leftarrow \theta^{n-1}$
        **end if**
    **end for**
    **return** $\theta$
**end procedure**

## Multiple chains

One possible qualitative modification we described was running multiple chains which swap positions stochastically. Each chain $c$ runs at different temperatures $\beta_c = 1/(1 + \Delta_T \times (c - 1))$, calculating the probability of a transition as $\min\{1, exp\{(\mathcal{L}(\theta') - \mathcal{L}(\theta_c^{n-1})) \times \beta_c\}\}$, where $\Delta_T$ is a free parameter that controls how steeply the temperature increases, and which we gave a uniform prior distribution, $\Delta T \sim U[1, 4]$. Then, in each iteration, after all chains have carried out both proposal and acceptance steps, a third swapping stage takes place: chains are randomly allocated to a pair, and for each pair, a position swap is proposed. These swaps are decided by comparing the joint likelihood of the current position-temperature pairs to what the joint likelihood would be if the chains swapped positions (lines 17-22 in Algorithm 2). If the swap is accepted, then the positions of the chains are exchanged. The number of chains to run is determined by a free parameter which we gave a uniform prior, $C \sim \{4, 5, 6\}$. Also, either all possible swaps or only one may be proposed at each iteration, which is determined by a third free parameter that we gave a uniform prior, $SA \sim \{0, 1\}$. Although when using this modification the position of the sampler is defined by a $N \times C$ matrix $\theta$ where each row is an iteration and each column one of the chains, only the positions of the cold chain ($\theta_{c=1}$) are returned.

## Gradient-based proposals

Another suggested modification to the Metropolis-Hastings proposal was to make proposals based on the gradient of the posterior distribution by simulating a physical system. Intuitively, algorithms using this modification will make proposals by simulating a frictionless particle in physical space, with the topology of the space being the negative log density (and thus its potential energy is proportional to that) and the kinetic momentum being drawn at random from a standard normal distribution at each iteration. Once

**Algorithm 2** Metropolis-coupled MCMC ($MC^3$)

1: **procedure** $MC^3(\theta^1, \mathcal{L}, N, \sigma, C, \Delta T, SA)$
2:     **if** SA = 1 **then** ▷ Initialize how many swaps will be attempted
3:         Set $nSwaps \leftarrow floor(C/2)$
4:     **else**
5:         Set $nSwaps \leftarrow 1$
6:     **end if**
7:     **for** c=1 to C **do**
8:         Set $\beta_c = 1/(1 + \Delta_T \times (c-1))$ ▷ Initialize Temperatures
9:     **end for**
10:     **for** n=2 to N **do**
11:         **for** c=1 to C **do** ▷ Each chain samples independently
12:             Set $\theta' \leftarrow \theta_c^{n-1} + N(0, \sigma)$
13:             Set $\alpha \leftarrow exp\{(\mathcal{L}(\theta') - \mathcal{L}(\theta_c^{n-1})) \times \beta_c\}$ ▷ $\alpha$ softened by the temperature
14:             Sample $u \sim U[0, 1]$
15:             **if** $u \leq \min\{1, \alpha\}$ **then** Set $\theta_c^n \leftarrow \theta'$ **else** Set $\theta_c^n \leftarrow \theta'$ **end if**
16:         **end for**
17:         **for** s=1 to nSwaps **do** ▷ Propose swaps after all chains have made a step
18:             Randomly select two chains $i, j$ without repetition
19:             Sample $u \sim U[0, 1]$
20:             Set $\alpha_{swap} \leftarrow \exp\{(\mathcal{L}(\theta_j^n) \times \beta_i) + (\mathcal{L}(\theta_i^n) \times \beta_j) - (\mathcal{L}(\theta_i^n) \times \beta_i) - (\mathcal{L}(\theta_j^n) \times \beta_j)\}$
21:             **if** $u \leq \min\{1, \alpha\}$ **then** Swap $\theta_i^n$ with $\theta_j^n$ **end if**
22:         **end for**
23:     **end for**
24:     **return** $\theta_{c=1}$ ▷ Only consider the cold chain
25: **end procedure**

the momentum has been initialized, the trajectory is simulated for a pre-set period of time, at which point the ending location is recorded as the new proposal.

In practice, the physical simulation needs to be carried out in discrete steps, by using a small step-size $\epsilon$ and approximately computing the position at times $\epsilon, 2\epsilon, \ldots, L\epsilon$ (a procedure for simulating the Hamiltonian equations while minimizing error is called the "leapfrog" method, which is shown in Algorithm 3). For this reason, the gradient-based samplers will use a measure of how long this trajectory will be simulated for as the product of $L \times \epsilon$. In our case, we fixed $\epsilon = .1$ and had $L$ as free parameter with a uniform prior, $L \sim \{1, 2, ..., 100\}$.

Once a proposal has been made, the transition probability is determined by the joint density of the position-momentum pair (not just the density of the position), and so the proposal will be accepted with probability $\min\{1, \frac{exp\{\mathcal{L}(\theta') - \frac{1}{2}p' \cdot p'\}}{exp\{\mathcal{L}(\theta^{n-1}) - \frac{1}{2}p \cdot p\}}\}$. Notice that the last step of the leapfrog algorithm (line 8) is to negate the momentum $p'$, which makes the Markov chain reversible.

If the sampler ran multiple chains, the swapping procedure in Algorithm 2, lines 17-22 would follow. Note that the joint density, not the density alone, should be used to calculate whether a swap should occur, and that the momentum variable for the $i^{th}$ chain should be drawn from $p \sim N(0, 1/\beta_i)$, not $p \sim N(0, 1)$.

For more information on gradient-based proposals and the leapfrog method, see Neal [2] and Betancourt [3].

## Recycled momentum

The final modification we proposed was to recycle the momentum. A sampler using this approach uses one additional parameter, $\alpha$, which we sampled $\alpha \sim U[0, 1]$. Theoretically, this algorithm can be implemented with negative $\alpha$ values up to -1, but that would lead to anticorrelated behavior, so we excluded this here as successive human utterances were positively autocorrelated.

Algorithms with this modification simply make a slight modification to how the momentum is drawn (ln. 15 in Algorithm 3), so that

$$p \leftarrow \alpha \times p^{n-1} + (1 - \alpha^2)^{\frac{1}{2}} \times v; \qquad v \sim N(0, 1)$$

**Algorithm 3** Hamiltonian Monte Carlo

**procedure** LEAPFROG($\theta', p', \mathcal{L}, \epsilon, L$)
    $p' \leftarrow p' - \epsilon/2 \times \nabla\mathcal{L}(\theta')$ ▷ Start with half step
    **for** i=1 to L **do**
        $\theta' \leftarrow \theta' + \epsilon \times p'$
        **if** $i \neq L$ **then** $p' \leftarrow p' - \epsilon \times \nabla\mathcal{L}(\theta')$ **end if**
    **end for**
    $p' \leftarrow p' - \epsilon/2 \times \nabla\mathcal{L}(\theta')$ ▷ End with half step instead of full step
    $p' \leftarrow -1 \times p'$ ▷ Negate momentum to keep proposal symmetric
    **return** $\theta', p'$
**end procedure**

**procedure** HMC($\theta^0$, $\mathcal{L}$, $N$, $\epsilon$, $L$)
    **for** n=1 to N **do**
        $\theta' \leftarrow \theta^{n-1}$
        $p \sim N(0,1)$; $p' \leftarrow p$ ▷ Initialize Momentum and make a copy
        $\theta', p' \leftarrow Leapfrog(\theta', p', \mathcal{L}, \epsilon, L)$ ▷ Proposal simulating physical system
        $\alpha \leftarrow \min\{1, \frac{exp\{\mathcal{L}(\theta') - \frac{1}{2}p' \cdot p'\}}{exp\{\mathcal{L}(\theta^{n-1}) - \frac{1}{2}p \cdot p\}}\}$
        $u \sim U[0,1]$
        **if** $u \leq \alpha$ **then**
            $\theta^n \leftarrow \theta'$
        **else**
            $\theta^n \leftarrow \theta^{n-1}$
        **end if**
    **end for**
    **return** $\theta$
**end procedure**

where $p^{n-1}$ is the momentum at the end of the last iteration ($p'$). Because in the standard leapfrog algorithm the momentum is negated at the end, samplers with this modification will have to negate the momentum again (to undo the change of direction that changing the sign involves). In the first iteration of the sampler and after a chain has swapped position the momentum is not recycled but drawn from a standard normal distribution (as in HMC).

# References

1. Cooper RP. Executive Functions and the Generation of "Random" Sequential Responses: A Computational Account. Journal of Mathematical Psychology. 2016; 73:153–68. DOI: 10.1016/j.jmp.2016.06.002. Available from: https://linkinghub.elsevier.com/retrieve/pii/S0022249616300414 [Accessed on: 2020 Nov 23]

2. Neal RM. MCMC Using Hamiltonian Dynamics. *Handbook of Markov Chain Monte Carlo*. Ed. by Brooks S, Gelman A, Jones G and Meng XL. 1st. Chapman and Hall/CRC, 2011 :113–62. DOI: 10.1201/b10905. Available from: https://www.taylorfrancis.com/books/9781420079425 [Accessed on: 2021 Apr 18]

3. Betancourt M. A Conceptual Introduction to Hamiltonian Monte Carlo. 2018. arXiv: 1701.02434. Available from: http://arxiv.org/abs/1701.02434. preprint
