# Supplementary material for: Explaining the flaws in human random generation as local sampling with momentum
Source: PLoS Comput Biol. 2024 Jan 5;20(1):e1011739. doi: 10.1371/journal.pcbi.1011739 (PMC10796055; doi:10.1371/journal.pcbi.1011739)
Supplement: S6 Text — (PDF) [file pcbi.1011739.s006.pdf]

S6 Text Model comparison in the two-dimensional condition of Experiment 2

In the two-dimensional condition of Experiment 2, we compared the local sampling algorithms to *iid* sampling only, as the schema model cannot sample in two dimensions. Models behaved very similarly in this task, being able to replicate participants' values for *Shape*, *Adjacencies*, *Distances*, and *Turning Points*, but having too high values for *Repetitions* in all occasions (see Fig A).

Quantitatively (see Fig B), we found strong support for local sampling models over *iid* ($BF_{10} = 5 \times 10^{173}$), with all participants being better described by a local sampling model. When comparing the qualitative features of local sampling algorithms, we found evidence against models running multiple chains ($BF_{10} = 2.8 \times 10^{-29}$), against gradient-based samplers ($BF_{10} = 10^{-5}$), and in favor of recycled momentum ($BF_{10} = 3.8 \times 10^{43}$). We are, however, extremely cautious with these conclusions, as model recovery results were very poor for local sampling algorithms, which were misclassified 82.6% of the time on average.

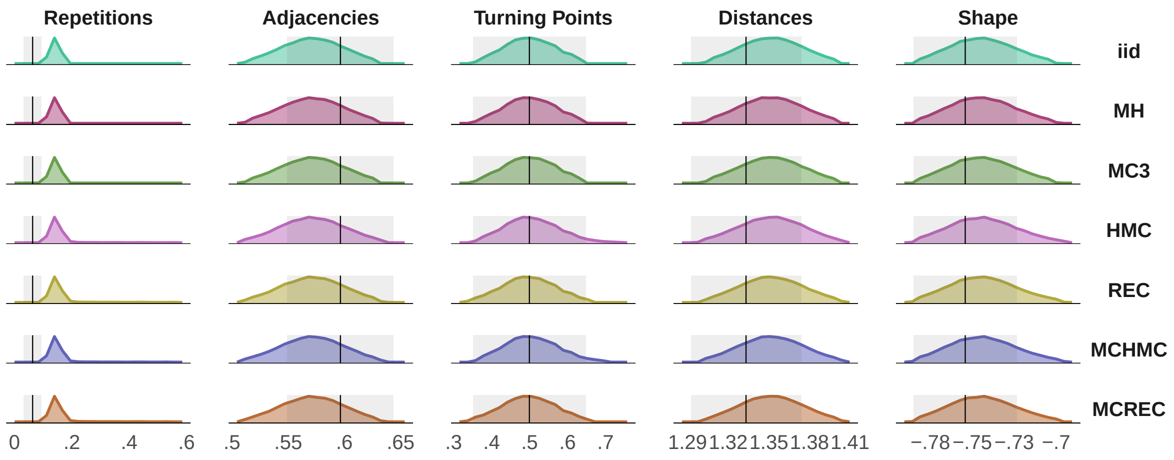

Fig A: Distribution of summary statistics per sampler in the two-dimensional condition of Experiment 2.

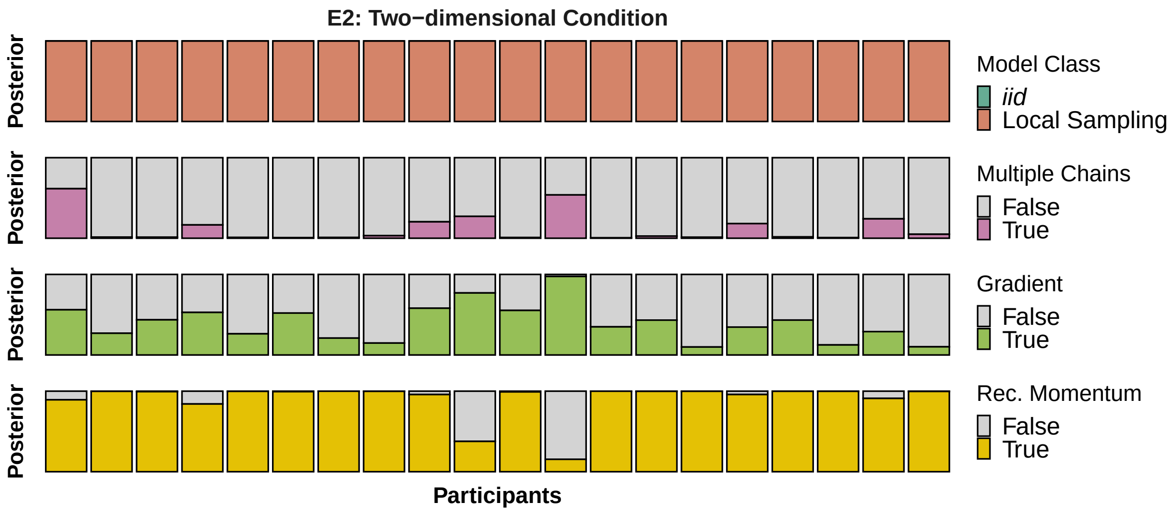

Fig B: Posteriors per participant in the two-dimensional condition of Experiment 2, with each column representing one participant. Local sampling algorithms replicated participants' data better than *iid* sampling. Evidence on qualitative features should be treated with skepticism, as model recovery results were poor for the local sampling algorithms.
