# Supplementary material for: Explaining the flaws in human random generation as local sampling with momentum
Source: PLoS Comput Biol. 2024 Jan 5;20(1):e1011739. doi: 10.1371/journal.pcbi.1011739 (PMC10796055; doi:10.1371/journal.pcbi.1011739)
Supplement: S7 Text — (PDF) [file pcbi.1011739.s007.pdf]

S7 Text Entropy Measures

In Cooper [1], the author uses two measures of entropy to evaluate the schema model. The first is called Redundancy, and measures entropy at the item level (i.e., whether all possible items are mentioned equally frequently). It is calculated as:

$$Red. = 1 - \frac{\log_2 n - \frac{1}{n}\sum_i n_i \times \log_2 n_i}{\log_2 a}$$

where n is the number of responses in the sequence, a is the number of alternatives, and n_i is the number of responses for the ith alternative. It ranges from 0 to 1, with 1 representing total predictability (always the same response), and 0 representing equal frequency of possible responses.

The second measure is Evans's [2] RNG measure, which captures the redundancy of bigrams:

$$RNG = \frac{\sum_{ij} n_{ij} \log_{10} n_{ij}}{\sum_i n_i \log_{10} n_i}$$

where n_i is the frequency of response i and n_{ij} is the frequency of response i followed by response j. It takes values of 0 when all possible bigrams appear equally in the sequence, and a value of 1 when each item is perfectly predicted by the one before it.

These measures are best used when the distribution is uniform, as it is only then where they represent departures from randomness (i.e. a set of random normally distributed variables should have some items and some bigrams appear more regularly than others). For this reason we did not carry out analyses using these measures in the Gaussian condition of Experiment 1.

S7.1 Results

As in previous literature, participants' Redundancy scores where larger than those expected from an *iid* sequence sampling from the same range (see Table A), for the uniform condition in Experiment 1 ($t(19) = 6.41, p < .001, BF_{10} = 1.2 \times 10^4$), and the one-dimensional condition ($t(39) = 3.6, p < .001, BF_{10} = 10$) and two-dimensional condition ($t(39) = 3.99, p < .001, BF_{10} = 22$) in Experiment 2. We found moderate evidence against a difference in Redundancy between the one and two-dimensional conditions in Experiment 2 ($t(38) = -1.22, p = .23, BF_{10} = 1/8$).

	Redundancy			RNG		
	Mean	SD	*iid*	Mean	SD	Reshuffled
E1 Unif	.12	.06	.06	.18	.14	.08
E2 1D	.02	.03	.004	.62	.11	.53
E2 2D	.01	.01	.005	.58	.05	.52

Table A: Participant's Redundancy and RNG measures, compared to the expectation from *iid* sampling or from reshuffling the sequence, respectively.

We also compared participants' RNG scores to those obtained from reshuffling their sequences 1000 times. Their RNG scores were larger than the expectation from reshuffled sequences in the uniform condition of Experiment 1 ($t(19) = 5.42, p < .001, BF_{10} = 530$), and the one-dimensional condition ($t(39) = 5.59, p < .001, BF_{10} = 975$) and two-dimensional condition ($t(39) = 8.08, p < .001, BF_{10} = 4.7 \times 10^6$) in Experiment 2. We found moderate evidence against a difference in RNG between the one and two-dimensional conditions in Experiment 2 ($t(38) = -1.48, p = .15, BF_{10} = 1/6$).

S7.2 Model Comparison

We carried out the same model comparison using ABC with random forests (as described in the main text), this time including Redundancy and RNG to the set of evaluated summaries. Qualitatively, our results are identical (see Table B): The best fitting model for the uniform condition was MCREC, and the best fitting model for the one-dimensional condition was the schema model. Using these updated posteriors also does not change conclusions for the qualitative features of the local sampling models, with Bayes Factors of inclusion supporting multiple chains ($BF_{10} = 4.0 \times 10^{25}$), gradient-based proposals ($BF_{10} = 1.5 \times 10^{69}$), and recycled momentum ($BF_{10} = 3.9^{41}$). As described in S6 Text, model recovery for the 2D condition was poor (error rate 82% among local sampling models), and so we are cautious drawing conclusions from those results.

Model	E1: Unif Condition	E2: 1D Condition	Combined Posteriors	E2: 2D Condition
MCREC	3.6×10^{95}	1.8×10^{49}	7.7×10^{250}	2.4×10^{178}
REC	1.0×10^{71}	2.2×10^{69}	1.9×10^{225}	1.1×10^{193}
MCHMC	9.9×10^{80}	7.5×10^{27}	2.0×10^{209}	6.3×10^{134}
Schema	8.8×10^{71}	1.5×10^{126}	7.2×10^{182}	NA
HMC	9.6×10^{54}	3.3×10^{41}	2.9×10^{180}	6.1×10^{132}
MC^3	4.7×10^{47}	4.1×10^{17}	1.4×10^{140}	1.3×10^{127}
MH	8.7×10^{-10}	7.7×10^{36}	4.0×10^{49}	1.3×10^{132}
iid	1	1	1	1

Table B: Model comparison results when including Redundancy and RNG to the set of considered summaries. Combined posteriors incorporate these new posteriors for the uniform condition in Experiment 1 and the one-dimensional condition in Experiment 2 into the already-existing posteriors for the Gaussian condition. Compare with Table 2

References

1. Cooper RP. Executive Functions and the Generation of "Random" Sequential Responses: A Computational Account. Journal of Mathematical Psychology. 2016; 73:153–68. DOI: 10.1016/j.jmp.2016.06.002. Available from: https://linkinghub.elsevier.com/retrieve/pii/S0022249616300414 [Accessed on: 2020 Nov 23]

2. Evans FJ. Monitoring Attention Deployment by Random Number Generation: An Index to Measure Subjective Randomness. Bulletin of the Psychonomic Society. 1978 Jul; 12:35–8. DOI: 10.3758/BF03329617. Available from: http://link.springer.com/10.3758/BF03329617 [Accessed on: 2021 Jan 11]
