# Supplementary material for: Explaining the flaws in human random generation as local sampling with momentum
Source: PLoS Comput Biol. 2024 Jan 5;20(1):e1011739. doi: 10.1371/journal.pcbi.1011739 (PMC10796055; doi:10.1371/journal.pcbi.1011739)
Supplement: S8 Text — (PDF) [file pcbi.1011739.s008.pdf]

S8 Text Standard Approximate Bayesian Computation

In addition to performing Approximate Bayesian Computation using random forests as reported in the main text, we also carried out ABC using a more 'traditional' approach. In standard ABC, simulated data is compared to observed data by computing the distance between their summary statistics (after these have been normalized in some way), then applying some sort of transformation so that pseudo-likelihoods are obtained from distances.

In our case, after generating one million sequences per candidate model and obtaining summary statistics for each of them, we standardized each summary statistic for every observed sequence i and simulated sequence j, as:

$$Z_{ij} = \frac{X - \mu_i}{\overline{\sigma_w}}$$

where μ_i is the observed sequence summary measure, and $\overline{\sigma_w}$ is the average of the within participants standard deviation for that measure. To calculate σ_w for each participant, we split the sequence into three subsequences of equal length, and obtained summary statistics for each. This way, for each simulated sequence j and observed sequence i there was a five-dimensional point X_{ij} with coordinates $(Z_R, Z_A, Z_{TP}, Z_D, Z_S)$, the origin point $O_i(0,0,0,0,0)$ defined the observed mean for participant i, and a unit increase in each dimension represented an increase in the size of $\overline{\sigma_w}$ for that dimension. Finally, we calculated a pseudo-likelihood for each observed-simulated sequence pair as

$$log(L_{ij}) = \frac{\rho(X_{ij}, O_i)}{-2 \times \delta^2}$$

where $\rho(\cdot)$ is the Euclidean distance, and δ is a steepness parameter that determines how quickly L_{ij} decreases as the numerator grows (This is fixed by hand, with the disadvantage that choosing too large a value leads to undiagnostic likelihoods, while choosing too small a value over-rewards simulations that had similar summaries to observed data by chance [as all models are stochastic]). This pseudolikelihood is proportional to the density of a multivariate Gaussian distribution with mean the between-participants average measures (μ_b) and a diagonal covariance matrix defined by the average of the within-participants standard deviation for the measures (σ_w). Here, we report results for $\delta = 1$, but show a wider range of steepness parameters in Table A.

The results using this approach are similar to those reported in the main text and S6 Text. In the One-dimensional condition, the schema model was superior to local sampling models ($BF_{10} = 2.4 \times 10^5$) and to *iid* sampling ($BF_{10} = 1.8 \times 10^4$) in replicating people's data. Although local sampling algorithms performed relatively similarly in this task, we still computed Bayes factors of inclusion for the three qualitative constituent features, finding evidence for multiple chains ($BF_{10} = 5$), against gradients ($BF_{10} = 1/292$), and no evidence for or against recycled momentum ($BF_{10} = 1.4$).

In the Uniform condition, we found support for local sampling algorithms over schema ($BF_{10} = 6.8 \times 10^{04}$) and *iid* ($BF_{10} = 2.1 \times 10^{15}$), and support for multiple chains ($BF_{10} = 94$) and gradients ($BF_{10} = 1.5 \times 10^{20}$), with no evidence for or against recycled momentum ($BF_{10} = 1/1.4$). This also took place in the Gaussian condition, with support for local sampling algorithms over schema ($BF_{10} = 5.2 \times 10^{09}$) and *iid* ($BF_{10} = 8.8 \times 10^{06}$), and support for multiple chains ($BF_{10} = 7.2$), and gradients ($BF_{10} = 5.7 \times 10^{04}$), and no evidence for or against recycled momentum ($BF_{10} = 1/3.4$).

Considering the joint posteriors across the three tasks, we find support for local sampling algorithms over schema ($BF_{10} = 1.4 \times 10^9$) and *iid* ($BF_{10} = 1.4 \times 10^{21}$), and support for multiple chains ($BF_{10} = 3.4 \times 10^3$), gradients ($BF_{10} = 2.9 \times 10^{22}$), and anecdotal evidence against recycled momentum ($BF_{10} = 1/3.46$). As described above, values of δ will influence this posterior, and so we show the joint posteriors over a range of values in Table A (with similar results).

Once more we computed the posterior for the two-dimensional condition separately, both because schema models cannot sample in two-dimensional space and because the task proved extremely undiagnostic. In this task, we found anecdotal evidence against local sampling against *iid* ($BF_{10} = 1/2.28$), no evidence for or against multiple chains ($BF_{10} = 1$), evidence against gradients ($BF_{10} = 4.27$) and inconclusive evidence regarding recycled momentum ($BF_{10} = 1/1.2$).

	$\delta = 0.8$	$\delta = 0.9$	$\delta = 1$	$\delta = 1.1$	$\delta = 1.2$
LS vs. Schema	1.08×10^{39}	2.24×10^{28}	1.37×10^{21}	1.19×10^{16}	2.53×10^{12}
LS vs. *iid*	6.36×10^{-4}	4.88×10^{4}	1.44×10^{9}	3.17×10^{11}	3.36×10^{12}
Multiple Chains vs not	2.73×10^{5}	1.86×10^{4}	3.40×10^{3}	1.08×10^{3}	4.65×10^{2}
Gradients vs not	4.52×10^{34}	5.52×10^{27}	2.93×10^{22}	2.68×10^{18}	1.98×10^{15}
Recycled Momentum vs not	3.29×10^{-1}	2.73×10^{-1}	2.89×10^{-1}	3.22×10^{-1}	3.62×10^{-1}

Table A: Joint posteriors across the Uniform and Gaussian conditions of Experiment 1 and the One-dimensional condition of Experiment 2, for several values of δ.
