# Supplementary material for: Explaining the flaws in human random generation as local sampling with momentum
Source: PLoS Comput Biol. 2024 Jan 5;20(1):e1011739. doi: 10.1371/journal.pcbi.1011739 (PMC10796055; doi:10.1371/journal.pcbi.1011739)
Supplement: S9 Text — (PDF) [file pcbi.1011739.s009.pdf]

S9 Text Exclusion Criteria

As reported in the main text, in Experiment 1 we calculated a measure of sequence determinism to identify participants who were not attempting to be random.

This measure, developed by Marwan et al. [1], is calculated by plotting a recurrent plot of the sequence, i.e. a plot showing when a sequence at time t is in the same position as a previous time (see Fig A). It is calculated by obtaining all diagonals and their lengths, and computing

$$DET = \frac{\sum_{l=2}^{l=N} lC(l)}{\sum_{1}^{N} lC(l)} \tag{1}$$

where l is the length of a diagonal, and $C(l)$ is the count diagonals of size l. For example, in Fig A there are 10 diagonals of length 1 (isolated squares), 4 diagonals of length 2, and the main diagonal has length 13. Determinism is thus:

$$DET = \frac{2 \times 4 + 13 \times 1}{10 \times 1 + 2 \times 4 + 13 \times 1} = .68$$

The average determinism for our participants was DET = .36, with SD = .12. One out of 21 participants was excluded following this criterion. The participant excluded from analysis had DET = .98. We reproduce their sequence below (in inches):

58, 59, 60, 60, 61, 62, 63, 64, 65, 66, 67, 68, 69, 70, 71, 72, 73, 74, 75, 76, 77, 78, 58, 59, 60, 61, 62, 63, 64, 65, 66, 67, 68, 69, 70, 71, 72, 73, 74, 75, 76, 77, 78, 58, 59, 60, 61, 62, 63, 64, 65, 66, 67, 68, 69, 70, 71, 72, 73, 74, 75, 76, 77, 78, 58, 59, 70, 61, 62, 63, 64, 65, 66, 67, 68, 69, 70, 71, 72, 73, 74, 75, 76, 77, 78, 58, 59, 60, 61, 62, 63, 64, 65, 66, 67, 68, 69, 70, 71, 72, 74, 75, 76, 78, 58, 59, 60, 61, 62, 63, 64, 65, 66, 67, 68, 69, 70, 71, 72, 73, 74, 75, 76, 77, 78, 58, 59, 60, 61, 62, 63, 64, 65, 66, 67, 68, 69, 70, 71, 72, 73, 74, 75, 76, 77, 78, 58, 59, 60, 61, 62

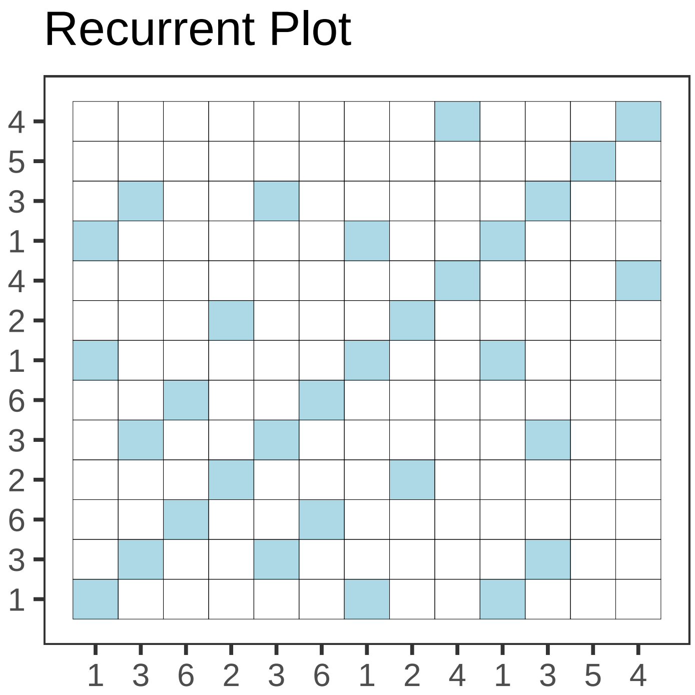

Fig A: Recurrent plot for an example sequence *1,3,6,2,3,6,1,2,4,1,3,5,4*.

In Experiment 2 we only excluded participants who could not prove that they had learned the display after four attempts or within 10 minutes (2 out of 42 participants).

References

1. Marwan N, Romano MC, Thiel M and Kurths J. Recurrence Plots for the Analysis of Complex Systems. Physics reports. 2007; 438:237–329
