# Supplementary material for: Explaining the flaws in human random generation as local sampling with momentum
Source: PLoS Comput Biol. 2024 Jan 5;20(1):e1011739. doi: 10.1371/journal.pcbi.1011739 (PMC10796055; doi:10.1371/journal.pcbi.1011739)
Supplement: Attachment — Submitted filename: 1020response.pdf [file pcbi.1011739.s011.pdf]

Dear Drs. Beierholm and Marinazzo,

Thank you for the opportunity to revise our work. We feel that our paper has benefited greatly from the changes that we have made in response to yours and the reviewers' comments. Thanks so much for your valuable input.

In particular, the paper now has a more cohesive and understandable narrative, and links to previous literature on random generation more explicitly. For example, following a reviewer's suggestion, we now also link to another important random generation model and evaluate our results in light of this account (although it has not yet been implemented as a computational model). We also make links in the discussion to the random generation literature on neurodiverse participants, and point to some of the many manipulations with neurotypical adults that have been previously been explored. We have included additional analyses and plots in the appendices that further explore the data (especially from Experiment 1), and added supplementary material to make the manuscript more self-contained. We have also made changes to the analysis code to ensure that it is easily reproducible by anyone who would like to explore this approach further. Finally, following PLOS guidelines, we have included figure-generating data to the supplementary materials (https://osf.io/dw8ez/).

We would like to thank the reviewers for the time and expertise they have invested. Below, we list each reviewer's comment and reply with how our paper has changed in response. For ease of reference, we give each paragraph a label, and as much as possible refer to which lines in the marked-up copy of the revised manuscript the changes we mention appear (in the marked-up copy, additions are blue and deletions red).

Best,

Lucas Castillo

Pablo León-Villagrá

Nick Chater

Adam Sanborn

REVIEWER #1

***R1P1**. This is an interesting paper, and the authors are no doubt correct in their claim that Cooper (2016, or actually, Cooper 2016 and Sexton & Cooper, 2014) provides the only computationally specified model of human random generation (RG) as a task in itself. Specifically, Cooper - and his predecessors in verbal theorising - looks at random number and random letter generation where human participants are instructed to make their responses "as random as possible" (Cooper et al., 2012) or to imagine drawing numbers/letters from a bag with replacement (Baddeley et al. 1986). Implicit in these cases is the assumption that sampling is from a uniform distribution (Baddeley et al (1998) specify something about the nature of sampling but Cooper et al. (2012) do not). Theorising in both papers (and many other papers on human RG) also incorporates sets of ordered stimuli, with the assumption that humans will need to inhibit the automatic activation of the next item in an overlearned sequence in order to produce something approaching random behaviour. This is all summarised by the current authors, and finding a more general account of random generation which covers not only this situation but others is an ambitious idea. Technically, I see nothing wrong with the work but it does raise a number of issues that (to my mind anyway) the current authors seem to skirt round rather than address head-on.*

Thanks for pointing out that Sexton & Cooper (2014) is the computational model of human random generation (RG) from which Cooper (2016) is derived. We have made this explicit when describing Cooper's (2016) model in the Model Comparison section (lines 280-281 and 287). It is also correct that stimuli in many RG papers have ordered stimuli, which we now mention in the Introduction (line 108).

***R1P2**. For example it seems as though Cooper's schema model has less breadth of coverage than the current authors' local sampling idea but is a more in-depth model within its own domain of coverage and this could be considered more thoroughly. Experiment 1 of the current study asks participants to randomly generate numbers either from a uniform or non-uniform distribution (this is novel to me, and interesting in itself) whereas Experiment 2 asked participants to generate items from a learned set. The authors select four measures of deviation (Repetitions, Turning Point, Adjacencies, (Euclidian) Distances) from serial independence on the pragmatic grounds that these can easily be applied to non-uniform distributions. However Cooper's (2016) model – and previous verbal accounts of experimental data – was developed to account for RG performance where the underlying distribution from which items are sampled might a priori be expected to be uniform and considers an overlapping but larger set of measures (Repetitions, Turning Point, Adjacencies, Bigram usage, Response Redundancies, Opposite Responses). Given the limited applicability of Cooper's model, why not look at some of these other measures in the conditions where the distribution is deliberately set up to be uniform? If Cooper's schema model eschews breadth for depth within a more limited set of circumstances it should outperform the authors' preferred sampling model and most other alternatives. Alternatively, if the schema model doesn't gain anything over the broader (sampling-based) alternatives even here then that would be good a priori reason to prefer the more general approach.*

Thanks for this suggestion. We re-did the model comparison using Cooper's Response Redundancies and Bigram Usage measures, with no qualitative change in the results. We describe these in the Supplementary Information S1. We also carry out standard analyses

comparing participants' data to what would be expected from true random generation, finding, like previous literature, that people are much worse at keeping the distribution of unigrams and bigrams flat (we show this in the Supplementary Information [Text G], and point to it in the main text in lines 330-333). We include code to calculate these measures to our uploaded code file. The other measure Cooper uses is a proportion of opposite responses. This can be calculated in his data, as there participants sample from a circular display, where every item has an opposite (similar to how numbers 1 and 7 oppose each other in a clock), but cannot be used here as our domains do not have natural opposites.

R1P3. *Similarly, for Experiment 2, the "generous assumption" (p. 7) is made that the schemata which Cooper's model relies upon can be quickly generated in order to apply to the briefly-learned stimuli used in this study although the current authors accept that the model cannot be applied to the two-dimensional condition of this experiment. I would be very surprised if appropriate schemata could be (over)learned this quickly. The whole point of a schema is that it is a powerful representational structure capable of dominating action unless actively suppressed or otherwise countered in some way. As such, the application of the schema model to these data seems like something of a straw man – I can't see any particular reason why it should be expected to work a priori (and this is neither a defence or a criticism of the model, simply an acceptance of its limitations).*

We have now expanded the discussion to explain that the reason this assumption is made is to be able to compare the local sampling account with another computational model of random generation (lines 292-294).We wouldn't characterise this assumption as a straw man, as it makes the schema model stronger, not weaker, allowing it to operate in situations where in theory it shouldn't be able to. In addition, the task where this assumption is made (one-dimensional condition of experiment 2) is the one task where the schema model outperforms the local sampling models.

R1P4. *Where it seems as though the schema model might have an edge is in potential applicability to RG tasks as undertaken by participants other than neurotypical young adults with no known brain damage or other impairment. The RG task was originally devised with this population (Baddeley, 1966), but it has typically been employed in subsequent studies as a means of assessing controlled processing, either testing for the presence of clinical impairment or to determine the cognitive demands of other, concurrently presented tasks (e.g., Jahanshahi et al., 2006). The authors recognise this and make brief reference to it (e.g., p. 1, p. 2) and the application of the schema model to such situations is conceptually transparent but it is less obvious how to apply the local sampling model to address these situations in a plausible manner. I have no doubt it can be done, the question is how to do it in a way that maintains a link with the (impaired) process being modelled rather than simply modelling the data and some more concrete suggestions along these lines would be welcome.*

Thanks for this suggestion. While our initial submission briefly mentioned how the local sampling models could be applied to neurodiverse populations, we have now expanded this and moved the paragraph to the Limitations and Future Directions section of the Discussion (lines 509-520). In short, much like different behavioural patterns could be replicated by different parameterizations of the schema model, so can they be replicated by different parameterizations, or different qualitative features, of a local sampling model. For example, an increased tendency to say adjacent items (like in schizophrenia; Salamé & Danion, 2007) can result from a change in the step size parameter of the local sampler or from not using

multiple chains, while a further decreased tendency to make a turn (like in multiple sclerosis; Geisseler et al., 2016) can be achieved by increasing the degree of recycled momentum. As we hypothesise that these sampling algorithms underlie behaviour in a wide range of tasks, this has potential implications for further understanding neurodiverse populations. However, this is of course outside of the scope of the current work, and modelling the different behavioural patterns found in different populations is potentially a very promising area for future research.

***R1P5**. The other aspect of this paper is the flip-side of this: the common use of a random element in models of human cognition which are not intended as models of RG per se. Here, the comparison is made between the local sampling approach and models incorporating "iid" (independent, identically distributed) sampling algorithms to generate the "noisiness" frequently observed in human behaviour. Local sampling models have already been compared to iid processes by some of the same authors elsewhere (e.g., Sanborn et al., 2022) so the work here extends this comparison specifically to the generation of sequences. I'm less familiar with the literature the authors cite for general comparisons between local sampling and iid processes but here the authors seem, in places, to conflate the scientific usefulness of a particular task as a method of investigation with the usefulness of the underlying process or sampling algorithm used to model performance on that task. For example, stating that in comparison with schema accounts " …the local sampling account is much more general, suggesting that people can draw samples from any probability distribution, including non-uniform distributions or multivariate distributions." (p. 2). This mixes up two different things. As the authors show in their experiments, their participants were perfectly capable of sampling from non-uniform distributions. This is an interesting idea as a potential new technique but the usefulness of this technique beyond the standard RG task has not so far been demonstrated and the mere fact that participants were able to follow instructions to do so at least as well (or as badly) as sampling from a uniform distribution is trivial in itself. Crucially it is not the local sampling account which suggests people can do this – a local sampling algorithm merely provides a better account of how people do this than iid sampling, and given the authors' previous work this is perhaps not a surprise.*

Apologies for the unclarity here. We have reworded the sentence the reviewer quotes to read "...the local sampling account is much more general, predicting that people will be able to draw samples from any distribution while matching their probabilities, including non-uniform distributions or multivariate distributions", which we believe avoids conflating the usefulness of the task and the usefulness of the proposed model (lines 111-118). We have also expanded on the fact that what we would expect is not only that participants can generate random items from a normal distribution, but that they do so while still displaying the same typical departures from randomness seen in the typical uniform task, which points to a common mechanism underlying their performance in this and other random generation tasks (lines 128-131). Although it is true that the fact that people can attempt to draw samples from non-uniform distributions is trivial, it is not trivial at all to find that they can do so while matching the expected densities -- in fact no existing model of random generation would have predicted this. The usefulness of this technique comes from this fact: that it can differentiate between different computational accounts of random generation better than sampling from a uniform distribution would (as evidenced by the large differences in the Shape measure in Experiment 1, for example, between the schema model and what participants and local sampling models do; see Figure 4).

R1P6. *In sum, I found this paper technically sound and it presents some interesting new ideas but the way in which these ideas are framed is not straightforward and sometimes a little confused (and confusing) such that the contribution of the approach within this area and its specific advantages over the existing accounts is not, ultimately, as obvious as I would have liked to see.*

Thanks, we have taken the opportunity to expand and clarify the framing both in the introduction and the discussion and believe that the result is now much clearer. We hope we now show that the present approach (1) tests two sets of differential predictions (Exp 1: non-uniform items; Exp 2: novel items) between a local sampling account and previous random generation accounts, and (2) shows that in these novel tasks participants are not only able to generate items at random, but that they do so while matching the relative probabilities of items and while showcasing the same deviations from iid than they do in the more typical random generation task. The fact that the same deviations from iid are shown here points to a common mechanism across all random generation tasks. (3) Thanks to this reviewer's suggestion, we now also show that in Experiment 1 this is not due to a separate set of measures being used to those in Cooper (2016) -- using those in our model comparison does not qualitatively change the results, showing that the local sampling model is both better at fitting the data overall and more general in its application. We also show that (4) the random generation task has great diagnostic power to identify qualitatively different models -- we identify several features of a local sampling algorithm that are best at reproducing the data, some of which such as "recycled momentum" had not been compared to people's performance previously.

REVIEWER #2

R2P1. *Summary*

======

This manuscript is part of a larger effort by the authors to account for systematic deviations from what might be expected from a more perfect cognitive system by postulating that the brain samples locally. Using that approach, they strive to reconcile what might be important, though divergent, models of cognitive function (e.g., Chater et al., 2020, for example). In this manuscript they take aim at random-pattern generation, striving to explain both why humans act more randomly than they should when they need not be random and why they are not random enough when specifically asked to be random. In particular, the authors claim that their model is better than the schema theory that, they say, currently prevails as an explanation for human random-pattern generation. The authors carry out two experiments, one of which was previously reported, to test this hypothesis. And they show that their results favor their model of local sampling with momentum. The application of their model to random-number generation is interesting and potentially fruitful. And the authors generally provide good evidence for it (though see below). But the manuscript suffers from several problems that are articulated below.

Thanks for this helpful high-level summary of the work.

R2P2. *The main drive of this study is pitting the authors' theory of random number generation against the schema theory, suggested by (Baddeley, 1996) and formalized by Cooper (2016).*

However, this theory is not as central in the field as the authors make it seem. In fact, many of the papers that they cite when discussing results in the field of random-number generation do not even mention schema theory. What is more, other models for random-number generation have been proposed, like Jahanshahi's more brain-based Network-Modulation Model, which the authors do not mention. So, aiming to provide evidence specifically against the schema theory to promote their own might be somewhat of a strawman argument.

> Thanks for pointing us to the network modulation model. While no computational model is available for this approach, so that cannot feature in our model comparison, it is important to include it in the introduction and discussion, discussing the current findings qualitatively in light of this model. We have now done so, adding paragraphs both in the introduction (lines 72-77) and the discussion (lines 426-441) discussing this model. We also clarify in the introduction why this model does not feature in our model comparison (lines 145-146). We have also modified, where applicable, mentions to the schema account to say "a popular account" instead of "the most popular account".

***R2P3**. What is more, it is unclear how strong that evidence is, or, in other words, whether the final conclusions about the schema vs MC samplers are warranted. This is because the schema model outperformed the other MC samplers on Experiment 2's one-dimensional data. Maybe adding a discussion on why MCREC did better on the aggregate data but not the individual datasets could help here.*

> We now clarify in the discussion (line 455) that MCREC did better on two of the three individual datasets reported in the main text, in addition to performing best on the aggregate data. We also now discuss why the schema model might have performed better in the one-dimensional condition of experiment 2 (lines 450-455).

***R2P4a**. In Experiment 1, the authors note that they chose to test the participants on a Gaussian (or normal) distribution rather than a uniform one, because that is a key differentiating factor between schema theory and theirs (lines 81-90 and 175-176). However, they could do more to explain why the schema theory does not allow for non-uniform distributions. While their claim may well be true, some simple extensions of the schema theory could make it better fit non-uniform distributions too. In sum, they should do more to explain why schema theory does not fit with nonuniform distribution to make it easier for the reader to understand.*

> We are by no means convinced that these potential extensions would be simple, and we now explain why in our discussion (lines 419-425). We have also expanded in the introduction on why other theories would not allow for non-uniform distributions in their current form (lines 108-111).

***R2P4b**. What is more, they wanted to test random-series generation in non-uniform settings. However, the Gaussian distribution is rather similar to the uniform one; ~⅔ of the weight lies within 1 STD from the mean, ~95% lies within 2 STDs from the mean, and almost all of it lies within 3 STDs from the mean. The authors use measures to specifically compare Gaussian to uniform distributions (Fig. 2). But to better argue for the ability of humans to generalize to non-uniform distributions, and to potentially make a better case against schema theory (at least as they see it), the authors might have selected, for example, a bimodal distribution, where there is little weight near the mean.*

While there are similarities, we disagree that the Gaussian and uniform distributions are similar when applied as in this paper. We tested whether the Shape measure was able to differentiate between the two: we ran 10000 simulations where we sampled 96 items (the smallest sequence in our sample) from the best fitting uniform and the best fitting normal in the Gaussian condition of Experiment 1, and ran a Welch t test between the Shape scores of both sequences. We found that the Shape scores from uniform and normal samples were significantly different in 99.53% of the simulations. In the text we report this power analysis in comparison with our initially-preregistered one (see R2P5b below).

In related research (León-Villagrá et al., 2022), we have shown that people can generate random samples from skewed distributions (such as gross earnings from films), as well as match fine details of a distribution (such as the fact that some values are very unlikely in American football scores). We now note this in the discussion (lines 492-496).

***R2P5a**. The authors mention pre-registering their results for both experiments. However, scrutinizing their preregistrations suggests that they do not always follow the exact methods they preregistered, which is problematic. They cannot expect every reader to read the preregistration and compare it to what they did. We give two examples below.*

Thanks for pointing out these deviations. We have dealt with the examples the reviewer mentions (see below), but we note that there are no other deviations from the pre-registration in our analyses.

***R2P5b**. * Experiment 1 pre-registration noted it would use BIC values to see how many participant generated sequences from a target distribution (e.g. uniform) would be better fitted by the target distribution or not (uniform vs. Gaussian) and test that against chance. Did this turn into the "Shape" value reported in the manuscript (which dies overall seems like a better measure)? If so, please clarify how. It's not clear what the reason for the change was.*

We carried out a power analysis with both measures, and found that the Shape measure had 3 times less Type II error rate than our initially pre-registered measure; 0.47% instead of 1.46%. The Shape measure is also more interpretable, as results from different conditions can be compared (as in Figure 2B). We now report this when we introduce the Shape measure (lines 235-240).

***R2P5c**. * A "sequence determinism" based on repeated subsequence exclusion criteria was mentioned in the pre-registration. Please clarify whether this was implemented but no participants were excluded because of it, or if it was not implemented, why?*

Thanks, one participant was excluded due to this criterion. We have clarified this in the main text (lines 567-573), and include an additional appendix outlining the exclusion criteria for both experiments and how they were applied. (Text I in Supplementary Information S1).

***R2P6**. To their credit, the authors provide a related manuscript, Castillo et al. (2021), to aid with the review process. However, their Experiment 2 was already reported there. So, the new things in this manuscript are Experiment 1 and to the overall discussion. Naturally, this limits the novelty of their current manuscript.*

Castillo et al. (2021) is a very early version of this current paper, as it was a conference proceedings publication rather than an archival publication. In their Ethical Publishing Practice, PLOS says that "Prior publication of research as a thesis, presentation at medical or

scientific conferences, or posting on preprint servers will not preclude consideration of your manuscript.", while the Cognitive Science Society state in their policy that their conference proceedings "are not considered archival for purposes of publication in either of the two Cognitive Science Society journals.", and that "We have no formal agreements with any other journals or societies. However, it is our experience that most journals and societies adopt the same position to Proceedings papers as we do".

In addition to adding Experiment 1 and the discussion to this version of the manuscript, there are other novel elements. Neither the distributional sensitivity nor the turns at the centre analyses are present in Castillo et al. (2021), and the model comparison on the Experiment 2 data is also novel.

***R2P7**. A related issue is that the manuscript is not easy to read and understand in general. In particular, without reading Castillo et al. (2021), it was very difficult to understand what they did in Experiment 2. This puts an unreasonable onus on the reader to read another paper to understand this one. At a minimum, the authors should go over the manuscript carefully and revise it to read more comprehensively. The manuscript should also be self-contained, without having to read other papers and the preregistration to fully understand what it is about.*

Apologies for this. We have made several changes to make the text easier to understand, such as expanding the introduction and the discussion, adding more detail on the procedure prior to the results, and including the exclusion criteria in the Supplementary Information so that readers do not need to access the preregistration to understand why participants' data were excluded from analysis.

***R2P8**. As noted above, the author's main effort seems to be to apply their local-sampling approach to randomness, as a sort of "case study" for their theory. Unfortunately, this shows. The authors do not appear to be that familiar with the literature on randomness (see above). Too little of that literature is discussed in the introduction (which might be fine as shorter introductions are now preferred). But they also have a very short discussion, which barely relates their results to the rest of the literature. To give just a few examples, the literature on randomness discusses differences in random-number generation ability following different instructions (Guseva, Bogler, Allefeld, & Haynes, 2023), depending on whether the task involved competition with feedback (Budescu & Rapoport, 1992; Wong, Merholz, & Maoz, 2021), and following many other manipulations. Again, these are just a few examples. Does their model predict any of these findings? Does it have anything to say about Jahanshahi's model?*

Thank you for these very helpful pointers to the wider literature. We have now expanded our discussion to deal with some of the previous findings in the random generation literature more at depth, highlighting some where we can speculate about how current the local sampling models would reproduce the findings (lines 535-541) as well as others where we would anticipate more modelling work would be needed to explain the results (lines 521-534). As discussed above, we have now included discussion on Jahanshahi's network modulation model both in the introduction and discussion of our paper.

***R2P9**. Code reproducibility: Seems like the code should work but we could not compile it on our local computer due to missing dependencies. Could authors send a docker container or list what needs to be installed to run their provided code? This seems to be a prerequisite of the journal.*

Apologies for the difficulty in running the code. We find that it does run once the dependencies are installed, and we have now reuploaded the code with a Readme file that lists the dependencies and explains how they should be installed if necessary.

R2P10. *Minor comments*

===========

Please clarify whether the participant reshuffled sequences and shape measure used the sequences before or after removing repetitions.

We have now clarified that both the reshuffled sequences and the Shape measure were generated/calculated without removing repetitions (lines 192 and 234, respectively).

R2P11. *The authors write that they focused on deviations from serial independence, as they are more interpretable than compressibility measures and can easily be applied to non-uniform distributions. This seems like weak justification. First, non-compressibility is one (some might say "the") measure of randomness. Serial independence is another. They could provide further justification here.*

It is true that compressibility is a valuable measure of randomness. However, our objective is not to determine that people are less random than iid sampling -- that is already well-established -- but to find the ways in which people deviate from randomness in order to build cognitive models that replicate those patterns. Consider the sequences of toin cosses HTTHTHTHTH, HHTTTTHHHT and THTHTHTTTT. They are all very compressible, but only using measures other than compressibility would help us find the mechanism that generated the biased sequences (in this case a bias towards alternations, repetitions, and tails, respectively). We have reworded this point slightly in lines 167-169.

REVIEWER #3

R3P1 & R3P2. *The authors study how humans can generate random sequences and find that their sequence generation deviates from iid sampling. They conduct 2 online experiments, one which uses their implicit belief about heights and the other which uses a learned sequence eliminating any prior belief effects. They find a similar deviation from iid sampling across experiments and evidence consistent with earlier studies. Next, they perform a formal model comparison between schema-type models (proposed earlier) and local sampling models. They found support for the local sampling models in experiment 1 and the 2D condition in experiment 2 but not in the 1D condition of experiment 2. However, they found the local sampling models explained certain characteristics of the model better in the 1D condition. Overall on the population level across conditions, they found strong support for the recycled MCMC model.*

Overall, I think this paper is well written and tackles a specific problem for which they present empirical insights and perform sophisticated model comparisons. However, I have a few questions that a few questions/comments that I would like the authors to address. I apologize if any of the answers are already present in the text and I missed them. In such a case, highlighting them more would increase the readability of the paper

Thank you for this clear summary of the work and the valuable suggestions below.

***R3P3**. The key confound that worries me regarding the evaluation of different mechanisms is how well the fitted canonical distribution matches the implicit distribution from which the participants draw the random sequences. To address this there are several possible suggestions:*

(a)Perform an additional experiment where the participants are either asked to sketch out the 1D distribution after the sample draw or implicitly are asked questions that tell insights about the distribution (quantiles for eg.) to estimate the actual distribution

(b)Without collecting new data, it would be nice to see some QQ plot-type comparisons showing absolute goodness of fits of the fitted and data distribution

(c) Along the lines of the above, plotting the histogram of the CDF of the canonical distribution evaluated at each of the participants' samples would be useful to see. If the canonical distributions are a perfect match, this would be a uniform distribution (CDF of samples drawn from the PDF are uniformly distributed)

Thank you for these suggestions. We have included a QQ plot comparing the fitted and data distributions to Text A of the Supplementary Information S1. However, we note that having a fitted canonical distribution match the implicit distribution better would likely only improve the performance of iid and the local sampling models, as the schema model assumes uniformity and thus samples from the range of possible responses. This is because the Shape measure would more closely match participants values as the fit improved (we now note this in Text A3 of Supplementary Information S1). For this reason we chose to fit a canonical distribution that was adequate, but did not focus our efforts on making the fit extremely precise. Regarding point (a), we note that in separate related work (León-Villagrá et al., 2022) we have looked at whether random generation can be used as a belief elicitation method, and found that it is competitive with other techniques such as obtaining estimates of the deciles or asking for a set of conditional predictions (lines 491-496).

***R3P4**. Did you try a mixture of canonical distributions to see if the quality of fit was better?*

We imagine that a mixture of canonical distributions might fit some participants better, but as pointed above this would likely only improve the performance of iid and the local sampling models.

***R3P5**. The results report best-fit Beta distributions or normal distributions. But as I understand, different distributions were fit for each participant. So, is the reported value a mean? Expanding on the clarity here would be helpful.*

The reported value is indeed a mean of the best fitting parameters for each participant's sequence. We have now clarified this when describing the model comparison method (lines 321-323) and re-emphasize it when describing the model comparison results (lines 338-339, 359 and 375).

***R3P6**. Could you compute and add errorbars to the Fig. 2B (maybe through bootstrapping)*

We have updated figure 2 to include 95% confidence intervals obtained via bootstrapping, and updated the caption to reflect this. While correcting this figure we noticed that it previously included the one participant whose data had been excluded from analysis - we have amended this now (this error was exclusively in this figure: data from this participant was not used in any reported analysis).

R3P7. *How did the sample distributions change over the course of generation? A QQ plot (or equivalent) of the first half and second half would be useful to see*

They did not seem to change much. We include this in Text A of the Supplementary Information S1 exploring whether in Experiment 1 distributions change over the course of generation. We follow the reviewer's suggestion of plotting an empirical QQ plot of the first and second half, and complement this with 2-sample Kolmogorov-Smirnov Tests testing the null hypothesis that both halves come from the same distribution.

R3P8. *A slightly big-picture question is how the explicit random sequence generation compares the implicit samples that participants could generate during Bayesian inference (as in the papers the authors reference). There are problems with explicit questions as choice biases enter the decision process (see https://elifesciences.org/articles/71866 for an example of this). There is no easy way to answer this with the data collected but if new data collection was possible one potential way would be to add a loss function for which the samples are generated (say to estimate the 3 most common heights or so), how the samples differ depending on the loss function. If the samples differed, asking participants to generate random samples as in the current experiment may depend on the implicit loss function they generate the samples for thereby being different for each participant. This is a very broad question so even if new data collection to answer this is not possible, I would be interested to hear the authors' thoughts on this.*

Thanks for raising this interesting question. Research on how people forage for ideas before making a choice seems to suggest that saying what one is thinking out loud before making the decision makes little or no difference on the outcome of a choice (Hardisty et al., 2010), and that asking people to come up with items is not related to whether those items serve a particular purpose or not (similar to the loss function suggestion the reviewer makes; Mills & Phillips, 2023). We have added these points to our discussion (lines 461-473), though of course more work is needed to establish a strong link. If task-related differences in how samples are obtained are small or non-existent, then the random generation task proves an excellent task to dissociate between different sampling paradigms: as shown here it has great diagnostic power among qualitatively different features.

R3P9. *It would be useful to see an autocorrelation plot of the participants' samples and those generated by different schemes.*

We have added such plots for data in Experiment 1 for both the computational models and participants (Text A in Supplementary Information S1). We do not provide these for Experiment 2 because, in such a small range, they look flat throughout.
