# Supplementary material for: Explaining the flaws in human random generation as local sampling with momentum
Source: PLoS Comput Biol. 2024 Jan 5;20(1):e1011739. doi: 10.1371/journal.pcbi.1011739 (PMC10796055; doi:10.1371/journal.pcbi.1011739)
Supplement: Attachment — Submitted filename: Author Response.pdf [file pcbi.1011739.s012.pdf]

Dear Drs. Beierholm and Marinazzo,

Thank you again for the opportunity to revise our work, and we also thank the reviewers for their time invested. Below, we list each reviewer's remaining questions and reply with how our paper has changed in response.

For ease of reference, we refer to which lines in the marked-up copy of the revised manuscript the changes we mention appear (in the marked-up copy, additions are blue and deletions red). This time we also provide a marked-up copy of Supplementary Information 1

Best,

Lucas Castillo

Pablo León-Villagrá

Nick Chater

REVIEWER #1: REVIEW BY C. PHILIP BEAMAN

I previously considered the paper technically sound and I still think the same. The framing and positioning of the paper, and in particular the comparison with Cooper's earlier work which I previously queried, has improved. Inevitably there are still a few things in the presentation I might personally take issue with but I don't see anything here that precludes publication.

Thank you.

REVIEWER #2

We thank the authors for taking our comments seriously. We think that the current version of this manuscript is much improved from its previously submitted one. We have only 1 major remaining question.

Major

1. Thank you for clarifying that a participant's reshuffled sequence did not have repeated items removed. Since this is the case, can the authors further provide an explanation for why they remove repeated items to calculate adjacencies, turning points, and distances for each participant's sequence, but do not remove repeated items after reshuffling? I am a little confused because at first glance, it seems that a fair comparison would require one to conduct the exact same analysis steps to both the experimental condition (i.e., participant's sequence) and the control (i.e., the participant's shuffled sequence). Otherwise, it seems superfluous to make statements such as "Compared to their reshuffled sequences, 208 participants' values were lower for Repetitions (Observed = .015, Expected = .043, Z = −2.61, p = .009, 209 d = −0.67, BF10 = 4)," (lines 208-209). Is this difference not created by necessity, since repeated items are removed from a participant's original uttered sequence, but not the reshuffled ones? This point would apply to the repetitions measure, adjacencies measure (line 172-174 mention the removal), turning points measure (lines 181-182), and distance measure (lines 183-184).

We believe there has been a misunderstanding. As the reviewer rightly points out, the same analysis should be carried out to both the real observed sequences and the reshuffled sequences. This is exactly what we did. What we meant in our previous response is that the reshuffled sequences were generated before item removal, not that indices were calculated differently between the experimental and control conditions. For example, if the observed sequence was "1,3,2,2,1", we would calculate the values of adjacencies, turning points and distances for the participant after removing repetitions (i.e. on the sequence "1,3,2,1"). However, when generating a new, reshuffled sequence, we would reorder the sequence before item removal (e.g. resulting in "3, 2, 1, 1, 2"). If this new reordering had a repetition, this would be removed ("3, 2, 1, 2") when calculating those indices. We have reworded this point slightly to make it clearer (lines 188-190).

Minor

** Line 24: Sentence does not seem grammatical. Did the authors mean to write: "When explicitly asked to be random, people are not random enough."*

We have corrected this, thank you (line 24).

** Wong et al. (2021) is used as an example that human-attempted random sequences are more compressible than those generated by machines. But that paper demonstrates that humans' randomness is statistically indistinguishable from that of computers in a competitive environment with feedback (when measuring randomness with compressibility).*

> Wong et al. (2021) does have conditions where human-attempted random sequences are more compressible than those generated by machines, but we agree that, since this is not the main finding of the paper, its inclusion here is confusing. We have reworded this passage (lines 64-66) to emphasise that greater compressibility is typically but not always the case, and now precede the Wong et al. reference with a "c.f. ".

REVIEWER #3

I want to first thank the authors for performing the additional required analyses and addressing my comments. I have one concern that I would like the authors to address:

Given the QQ plot in A3, there seems to be systematic deviation for the empirical distribution for most participants as compared to the best bit underlying distributions. While I understand and accept that a better fit to the theoretical distribution would improve the shape value and provide stronger evidence in favor of the sampling models compared to the schema, it is unclear how these deviations manifest in the deviations from IID sampling and various local sampling models that the authors consider.

For eg. if the underlying distributions the participants were sampling from was flatter than the theoretical distribution (as would be evidenced from the flat part in the QQ plots seen in several participants), then the distances even for IID samplers would be smaller than what is used for comparison. The other metrics would also differ. So to address this:

1) The authors could either using a nonparametric approximation of the empirical distribution (binning finely) as the target distribution or use a mixture distribution to get a better match and perform the subsequent analyses with the better fit target distribution

2) Or if the above analysis is not feasible, then they could subselect the participants whose QQ plot suggest non-significant deviation from the assumed theoretical distribution and compute the measures for those participants (if sufficient in number to draw useful insights).

> This is a very good point: The reviewer is right that some measures other than Shape would also differ with the fit, which could reward or penalise some models. For feasibility, we have now carried out the reviewer's second suggestion: we conducted one-sample Kolmogorov-Smirnov tests to find which participants were not fit well, and we re-analysed our model comparison after excluding these participants from analysis. Our results do not change qualitatively. We report this is Text A3 of Supplementary Information 1. We also change the figure and its caption to highlight which sequences had a significant Kolmogorov-Smirnov statistic.
