# Supplementary material for: Explaining the flaws in human random generation as local sampling with momentum
Source: PLoS Comput Biol. 2024 Jan 5;20(1):e1011739. doi: 10.1371/journal.pcbi.1011739 (PMC10796055; doi:10.1371/journal.pcbi.1011739)
Supplement: Attachment — Submitted filename: Computational Biology RNG Review.docx [file pcbi.1011739.s010.docx]

Review by C. Philip Beaman

This is an interesting paper, and the authors are no doubt correct in their claim that Cooper (2016, or actually, Cooper 2016 and Sexton & Cooper, 2014) provides the only computationally specified model of human random generation (RG) as a task in itself. Specifically, Cooper - and his predecessors in verbal theorising - looks at random number and random letter generation where human participants are instructed to make their responses “as random as possible” (Cooper et al., 2012) or to imagine drawing numbers/letters from a bag with replacement (Baddeley et al. 1986). Implicit in these cases is the assumption that sampling is from a uniform distribution (Baddeley et al (1998) specify something about the nature of sampling but Cooper et al. (2012) do not). Theorising in both papers (and many other papers on human RG) also incorporates sets of ordered stimuli, with the assumption that humans will need to inhibit the automatic activation of the next item in an overlearned sequence in order to produce something approaching random behaviour. This is all summarised by the current authors, and finding a more general account of random generation which covers not only this situation but others is an ambitious idea. Technically, I see nothing wrong with the work but it does raise a number of issues that (to my mind anyway) the current authors seem to skirt round rather than address head-on.

For example it seems as though Cooper’s schema model has less breadth of coverage than the current authors’ local sampling idea but is a more in-depth model within its own domain of coverage and this could be considered more thoroughly. Experiment 1 of the current study asks participants to randomly generate numbers either from a uniform or non-uniform distribution (this is novel to me, and interesting in itself) whereas Experiment 2 asked participants to generate items from a learned set. The authors select four measures of deviation (Repetitions, Turning Point, Adjacencies, (Euclidian) Distances) from serial independence on the pragmatic grounds that these can easily be applied to non-uniform distributions. However Cooper’s (2016) model – and previous verbal accounts of experimental data – was developed to account for RG performance where the underlying distribution from which items are sampled might a priori be expected to be uniform and considers an overlapping but larger set of measures (Repetitions, Turning Point, Adjacencies, Bigram usage, Response Redundancies, Opposite Responses). Given the limited applicability of Cooper’s model, why not look at some of these other measures in the conditions where the distribution is deliberately set up to be uniform? If Cooper’s schema model eschews breadth for depth within a more limited set of circumstances it should outperform the authors’ preferred sampling model and most other alternatives. Alternatively, if the schema model doesn’t gain anything over the broader (sampling-based) alternatives even here then that would be good a priori reason to prefer the more general approach.

Similarly, for Experiment 2, the “generous assumption” (p. 7) is made that the schemata which Cooper’s model relies upon can be quickly generated in order to apply to the briefly-learned stimuli used in this study although the current authors accept that the model cannot be applied to the two-dimensional condition of this experiment. I would be very surprised if appropriate schemata could be (over)learned this quickly. The whole point of a schema is that it is a powerful representational structure capable of dominating action unless actively suppressed or otherwise countered in some way. As such, the application of the schema model to these data seems like something of a straw man – I can’t see any particular reason why it should be expected to work a priori (and this is neither a defence or a criticism of the model, simply an acceptance of its limitations).

Where it seems as though the schema model might have an edge is in potential applicability to RG tasks as undertaken by participants other than neurotypical young adults with no known brain damage or other impairment. The RG task was originally devised with this population (Baddeley, 1966), but it has typically been employed in subsequent studies as a means of assessing controlled processing, either testing for the presence of clinical impairment or to determine the cognitive demands of other, concurrently presented tasks (e.g., Jahanshahi et al., 2006). The authors recognise this and make brief reference to it (e.g., p. 1, p. 2) and the application of the schema model to such situations is conceptually transparent but it is less obvious how to apply the local sampling model to address these situations in a plausible manner. I have no doubt it can be done, the question is how to do it in a way that maintains a link with the (impaired) process being modelled rather than simply modelling the data and some more concrete suggestions along these lines would be welcome.

The other aspect of this paper is the flip-side of this: the common use of a random element in models of human cognition which are not intended as models of RG per se. Here, the comparison is made between the local sampling approach and models incorporating “iid” (independent, identically distributed) sampling algorithms to generate the “noisiness” frequently observed in human behaviour. Local sampling models have already been compared to iid processes by some of the same authors elsewhere (e.g., Sanborn et al., 2022) so the work here extends this comparison specifically to the generation of sequences. I’m less familiar with the literature the authors cite for general comparisons between local sampling and iid processes but here the authors seem, in places, to conflate the scientific usefulness of a particular task as a method of investigation with the usefulness of the underlying process or sampling algorithm used to model performance on that task. For example, stating that in comparison with schema accounts “ …the local sampling account is much more general, suggesting that people can draw samples from any probability distribution, including non-uniform distributions or multivariate distributions.” (p. 2). This mixes up two different things. As the authors show in their experiments, their participants were perfectly capable of sampling from non-uniform distributions. This is an interesting idea as a potential new technique but the usefulness of this technique beyond the standard RG task has not so far been demonstrated and the mere fact that participants were able to follow instructions to do so at least as well (or as badly) as sampling from a uniform distribution is trivial in itself. Crucially it is not the local sampling account which suggests people can do this – a local sampling algorithm merely provides a better account of how people do this than iid sampling, and given the authors’ previous work this is perhaps not a surprise.

In sum, I found this paper technically sound and it presents some interesting new ideas but the way in which these ideas are framed is not straightforward and sometimes a little confused (and confusing) such that the contribution of the approach within this area and its specific advantages over the existing accounts is not, ultimately, as obvious as I would have liked to see.
